# Supplementary material for: Integrated biosensor platform based on graphene transistor arrays for real-time high-accuracy ion sensing
Source: Nat Commun. 2022 Aug 27;13:5064. doi: 10.1038/s41467-022-32749-4 (PMC9420106; doi:10.1038/s41467-022-32749-4)
Supplement: Supplementary file 1 — Supplementary Information [file 41467_2022_32749_MOESM1_ESM.pdf]

Supplementary Information for

Integrated biosensor platform based on graphene transistor arrays for real-time high-accuracy ion sensing

Mantian Xue*‡[1], Charles Mackin‡[2], Wei-Hung Weng[1], Jiadi Zhu[1], Yiyue Luo[1], Shao-Xiong Lennon Luo[3], Ang-Yu Lu[1], Marek Hempel[1], Elaine McVay[1], Jing Kong[1], Tomás Palacios*[1]

[1]*Department of Electrical Engineering & Computer Science, Massachusetts Institute of Technology, Cambridge, USA.*

[2]*IBM Research–Almaden, 650 Harry Road, San Jose, CA, USA*

[3]*Department of Chemistry and Institute for Soldier Nanotechnologies, Massachusetts Institute of Technology, Cambridge, USA.*

* Correspondence author email: mxue@mit.edu (M.X.); tpalacios@mit.edu (T. P.)

‡These authors contributed equally to this work.

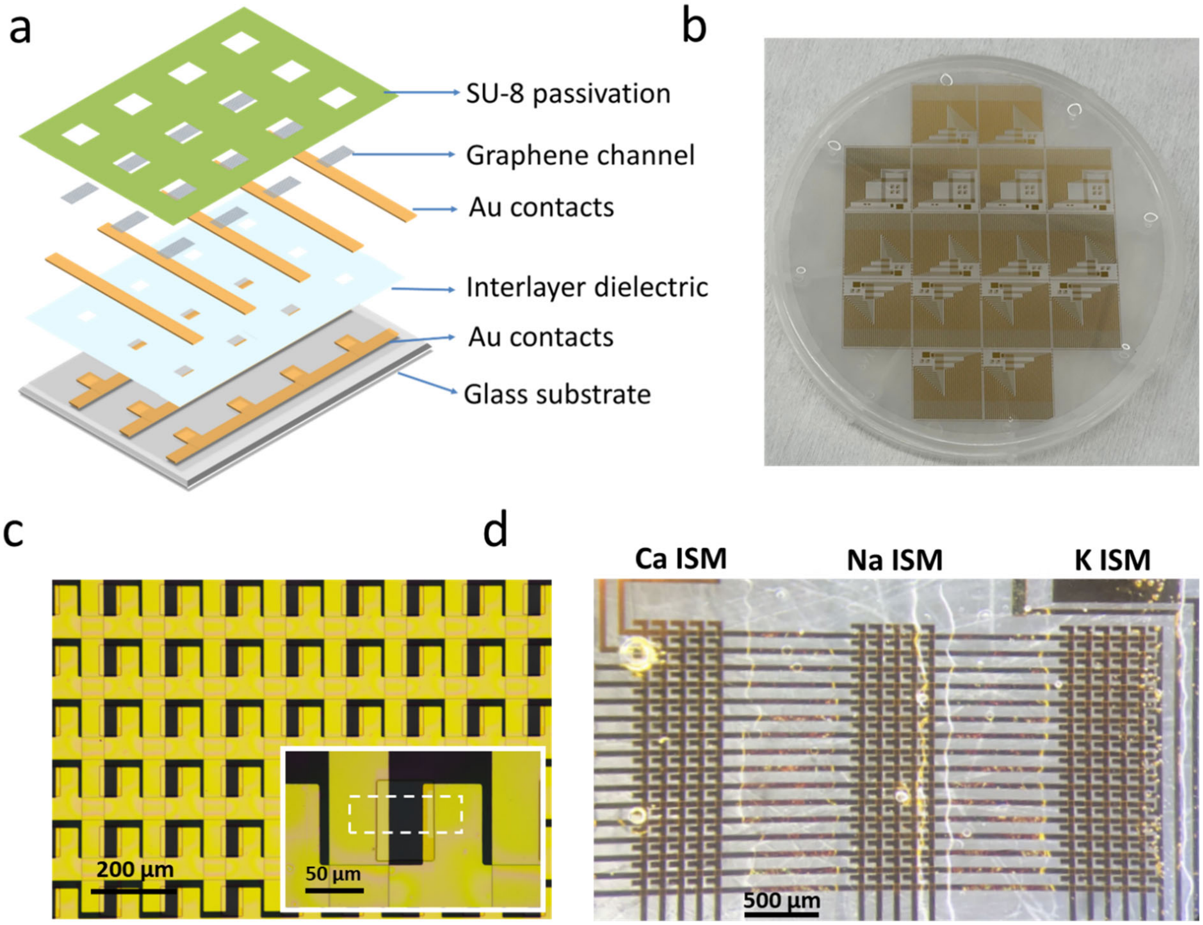

Supplementary Figure 1 | Device Geometry and Device Optical Image. a Graphene sensor chip fabrication layers. **b** Image of 16 sensing chips fabricated on a 4-inch wafer. **c** Optical image of the as-fabricated graphene sensor array. White rectangular box in the insert is the outline of a graphene channel. **d** integrated sensor array with Ca, Na and K ISM printed on the sensor array.

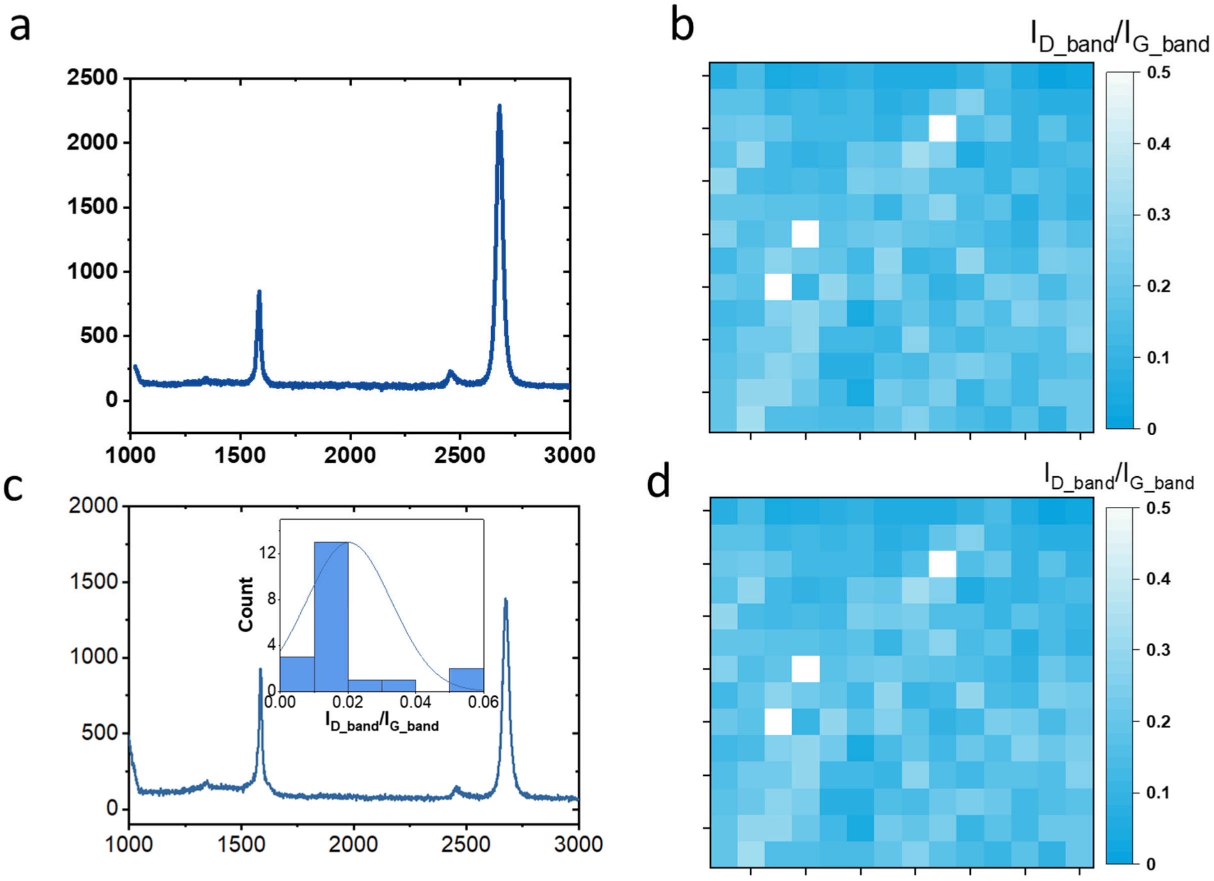

Supplementary Figure 2 | Raman Characterization. a Raman spectrum of the intrinsic graphene sheet before any fabrication. **b** *2D/G* ratio mapping of a 10mm-by-10mm graphene film before fabrication. **c** Raman spectrum of a graphene channel after the entire fabrication process. Insert is the histogram showing the distribution of the I_{D_band}/I_{G_band} ratio of graphene channels on the sensing array after fabrication. 20 devices were sampled on the same array. **d** I_{D_band}/I_{G_band} ratio map of a 20μm-by-20μm graphene channel after fabrication. The average of the I_{D_band}/I_{G_band} ratio is 0.129 with a standard deviation of 0.097.

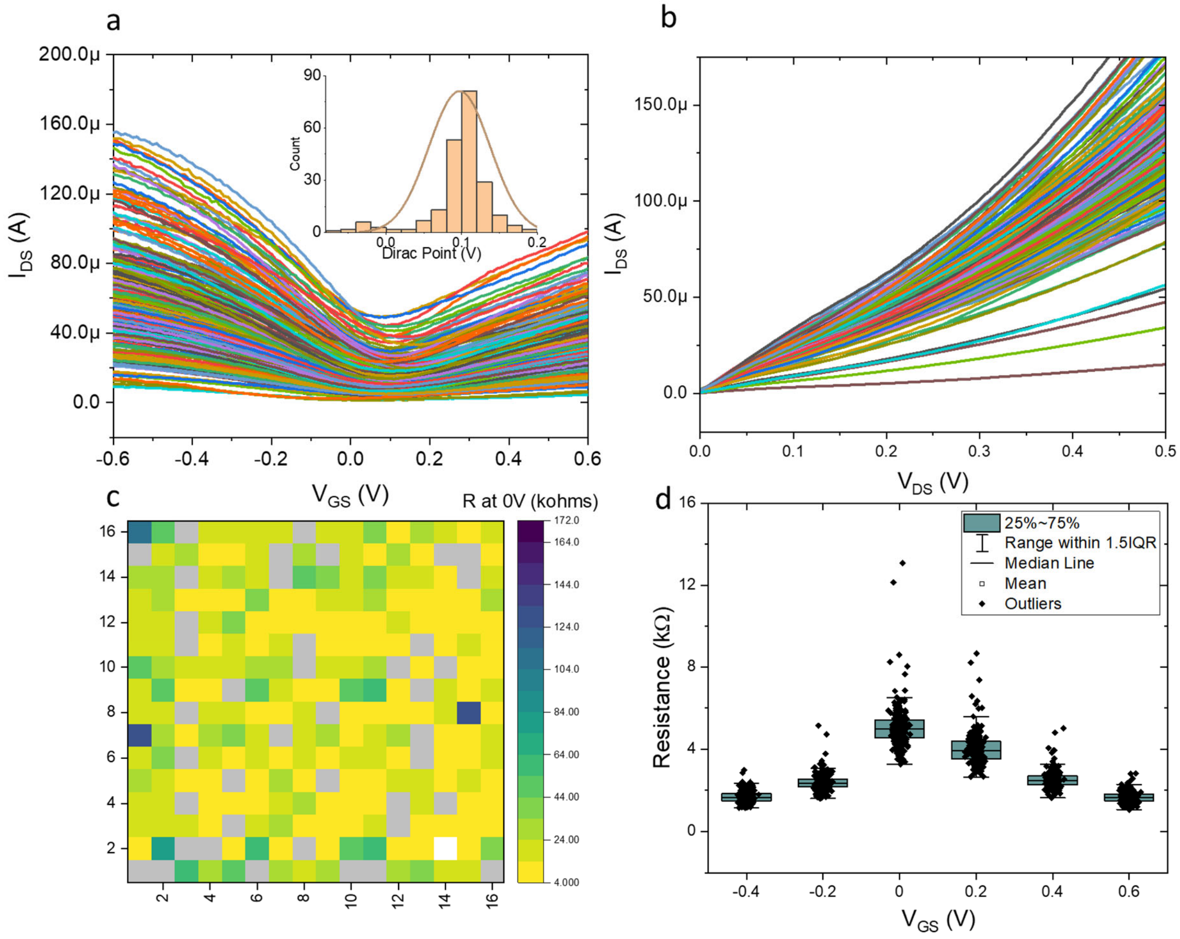

Supplementary Figure 3 | Variation in as-fabricated graphene sensor array. a I_{DS}-V_{GS} characteristics of an as-fabricated graphene sensing chip without functionalization. The sensing chip was tested in water with an Ag/AgCl reference electrode and biased at V_{DS} = 250 mV. Insert is the histogram of Dirac Points extracted from **a**. The average Dirac Point is a 97.1 mV ± 40.7 mV. **b** I_{DS}-V_{DS} characteristics of an as-fabricated graphene sensing chip without functionalization. Sensor chip is tested in water with zero gate bias. **c** color map of channel resistance for the whole array with V_{DS} =250mV and V_{GS}= 0. **d** graphene channel resistance distribution under various gating conditions.

Supplementary Note 1. ISM Operation Principle

The ion-selective membrane in this work is based on a charge neutral ionophore. Ionophores are lipophilic molecules that selectively bind to an ion of interest. Ionophores are especially useful in sensing applications because they provide both sensitivity and selectivity. In addition, because ionophores are lipophilic, they possess a high affinity for the membrane phase over the solution phase and may be assumed confined to the membrane. When a neutral ionophore is used, lipophilic ion sites with opposite charge of the analyte ion – in this case anionic sites -- have to be added in order to suppress the extraction of chlorite into the membrane [1–3]. Previous research also shows that these ionic sites in the membrane with an optimized ratio can effectively reduce response time, lower the electrical membrane resistance and increase selectivity [1].

In order to obtain electroneutrality inside the membrane, the concentration of cation must balance the total charges of the anionic sites. Since the number of anionic sites are fixed when preparing the membrane, concentration of target ion is constant within the membrane phase and is independent of concentration of the electrolyte outside of the membrane. This translates into an electrolyte-interface potential that is solely a function of the target analyte concentration. Because of this, the interface potential can be related back to the target analyte concentration in the electrolyte phase.

Using Ca^{2+} ion as an example, the potential at the membrane-electrolyte is governed by the Nernst equation as given by (1)

$$V_{ME} = V_M - V_E = V^0 + 2.3\frac{RT}{zF}\log\left(\frac{[Ca_E^{2+}]}{[Ca_M^{2+}]}\right), \qquad (1)$$

where V_{ME} is the potential difference between the membrane and electrolyte, V_E is the electrolyte potential, which equals to V_{GS} in Fig. 1a, V_M is the membrane potential, V^0 represents

all electrolyte-independent potential contributions. R is the gas constant, T is the temperature, z is the charge number of the analyte, F is the Faraday constant, $[Ca_E^{2+}]$ is the concentration of Ca^{2+} in the electrolyte phase, and $[Ca_M^{2+}]$ is the Ca^{2+} concentration present in the membrane phase. When $[Ca_M^{2+}]$ remains constant, the potential can be related directly to the concentration of $[Ca_E^{2+}]$ as shown in (2)

$$V_{ME} = V^{\theta} + 2.3\frac{RT}{zF}\log([Ca_E^{2+}]), \qquad (2)$$

where V^0 has been renamed V^{θ} to include the constant term resulting from the $\log([Ca_M^{2+}])$. For a bivalent ion such as Ca^{2+} at room temperature, the slope is theoretically approximately 30 mV/decade. For Na^+ and K^+ ion at room temperature, the theoretical slope is 60mV/decade. This translates directly to the minimum conduction point, or Dirac point, which shifts by the same amount according to (3)

$$V_{\text{Dirac}} = V_{\text{Dirac}}^0 - 2.3\frac{RT}{zF}([Ca_E^{2+}]). \qquad (3)$$

Because graphene EGFET have a V-shaped and approximately linear current-voltage *(I-V)* characteristic away from the minimum conduction point, there exists a direct linear relationship between the shift in voltage and change in current. Therefore, it is possible to relate the change in current to the change in analyte concentration as given by (4)

$$I_{DS} = I_{DS}^0 + k\log([Ca_E^{2+}]), \qquad (4)$$

where I_{DS}^0 is some constant baseline source-drain current and k is the slope of the voltage shift multiplied by the slope of the graphene *I-V* curve at that particular point.

In addition to the ideal electrostatic gating effect from the Nernst equation, defect sites on the graphene channel, including but not limited to grain boundaries, vacancies, contaminations,

substrate doping etc., can induce charge transfer effect to the graphene channel. Such effect will modulate the channel fermi-level hence induces an additional leakage current to the gate and shifts in the *I-V* characteristics[4–6].

Supplementary Note 2. Device Filtering Criteria

The non-functioning devices on the sensor array are filtered out before data analysis. The filtering conditions are the following:

- Filter out possible shorted channel: $I_{DS} > 170$ μA
- Filter out possible broken channel: $I_{DS} < 2$ μA
- Filter out abnormal channel:
    - Dirac Point not within the V_{GS} sweeping range (-0.6V – 0.9V)
    - $1 < I_{DS,max} / I_{DS.min} < 10$

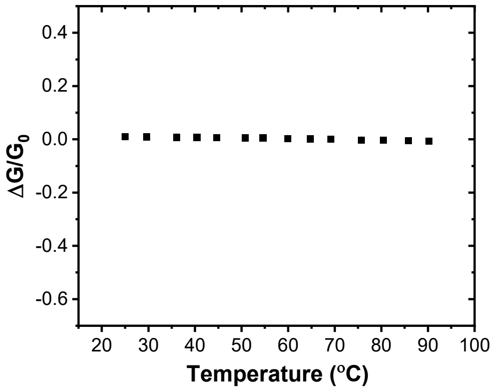

Supplementary Figure 4. Change in graphene conductance at different temperature.

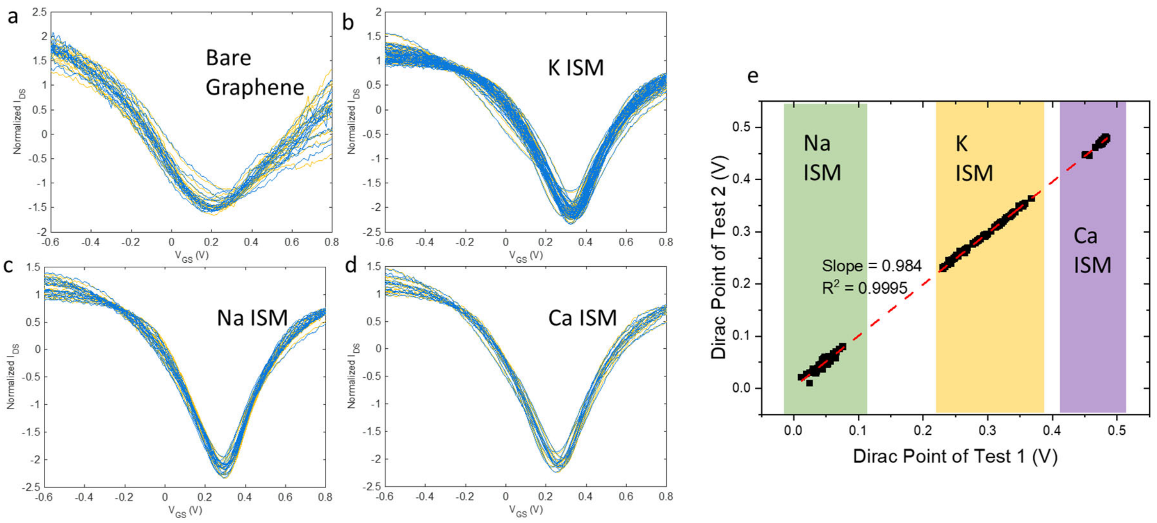

Supplementary Figure 5 | Reproducibility of IV measurement. Normalized *I-V* characterstics of two consecutive measumrents (yellow and blue curves) demonstrate reproducible and stable measuremnts of **a** 20 randomly selectived device from a graphene sensor array without functionalization; **b** sensors with K^+ ISM functionalization; **c** sensors with Na^+ ISM functionalization; and **d** sensors with Ca^{2+} ISM functionalization. e) Extracted Dirac points of two consecutive measumrents. Dirac points of test 1 (from yellow *I-V*s) are plotted as x-vlaues and that of test 2 (from blue *I-V*s) are plotted as y-values. A fitted line (red dash line) with a slope of 0.984 with an R-squared value of 0.999, indicating good agreement of the two measurements.

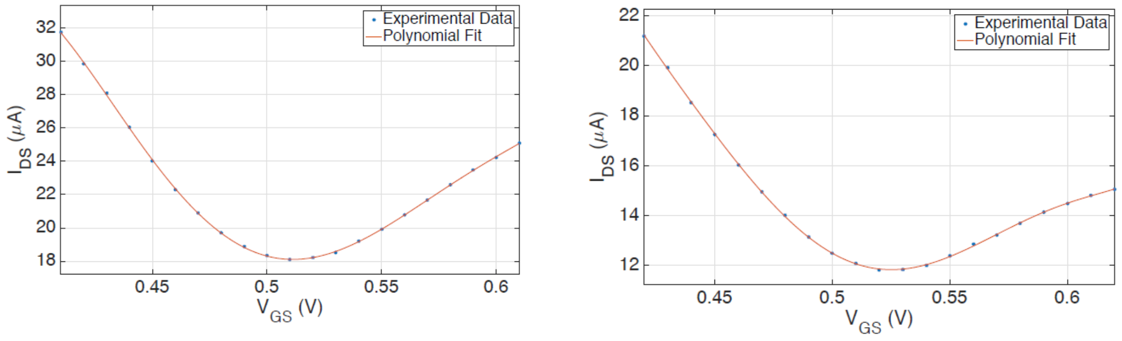

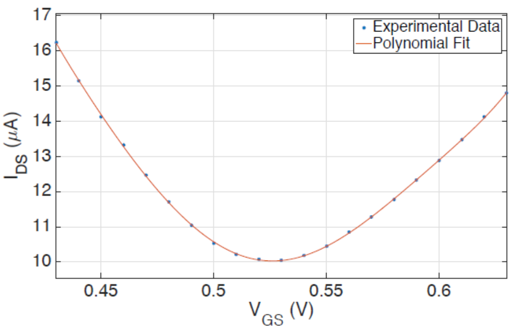

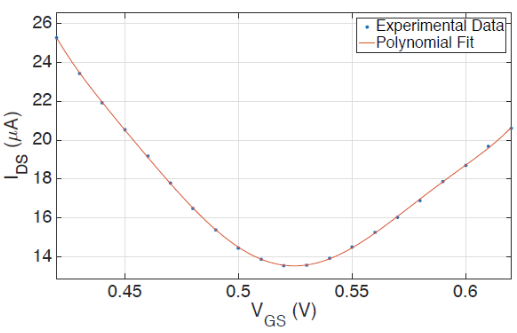

Supplementary Figure 6 | Dirac Point Estimation. Four examples showing how the Dirac points were estimated by fitting the discretized experimental *I-V* characteristic with a 6th-order polynomial and finding the minimum of the polynomial fit. This ensures the most accurate possible Dirac point interpolation.

Supplementary Note 3. Dirac Point Estimation

Minimum conduction points, or Dirac Points, were calculated by fitting the experimental *I-V* characteristic with a polynomial and finding the minimum of the polynomial fit. This ensures the most accurate possible Dirac point interpolation. *I-V* curves were fitted using a 6th-order polynomial. Examples in Supplementary Fig. 6 show that 6th-order polynomials fit the experimental data exceptionally well and that the Dirac points interpolated using this method are perfectly reasonable. Experimental data was discretized using 10 mV step size, which could lead to some additional error if the Dirac point was estimated simply by taking the minimum of the discretized dataset.

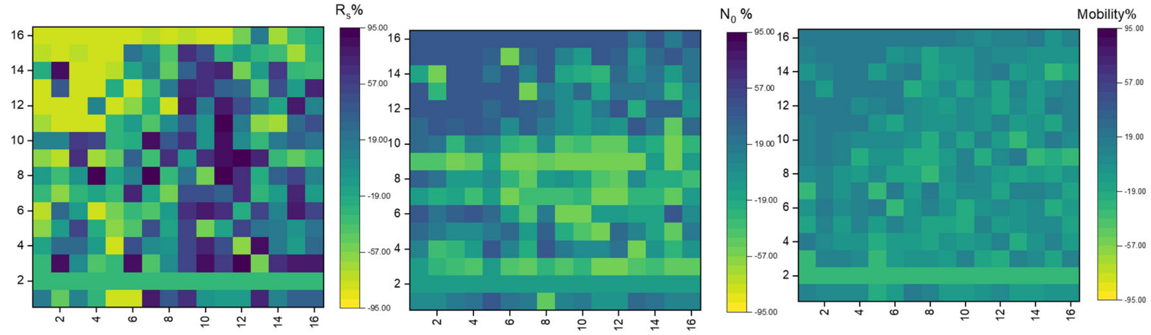

Supplementary Figure 7 | Extracted of parameters. Percentage change from average value of extracted contact resistance (left), carrier concentration (middle) and mobility (right) of Na^+ ISM functionalized sensor chip tested in 1 mM NaCl solution. The mean extracted values for contact resistance, carrier concentration and mobility are 260.9 kΩ µm, 2.1*10^{12} cm^{-2}, and 529.6 cm^2/V s respectively.

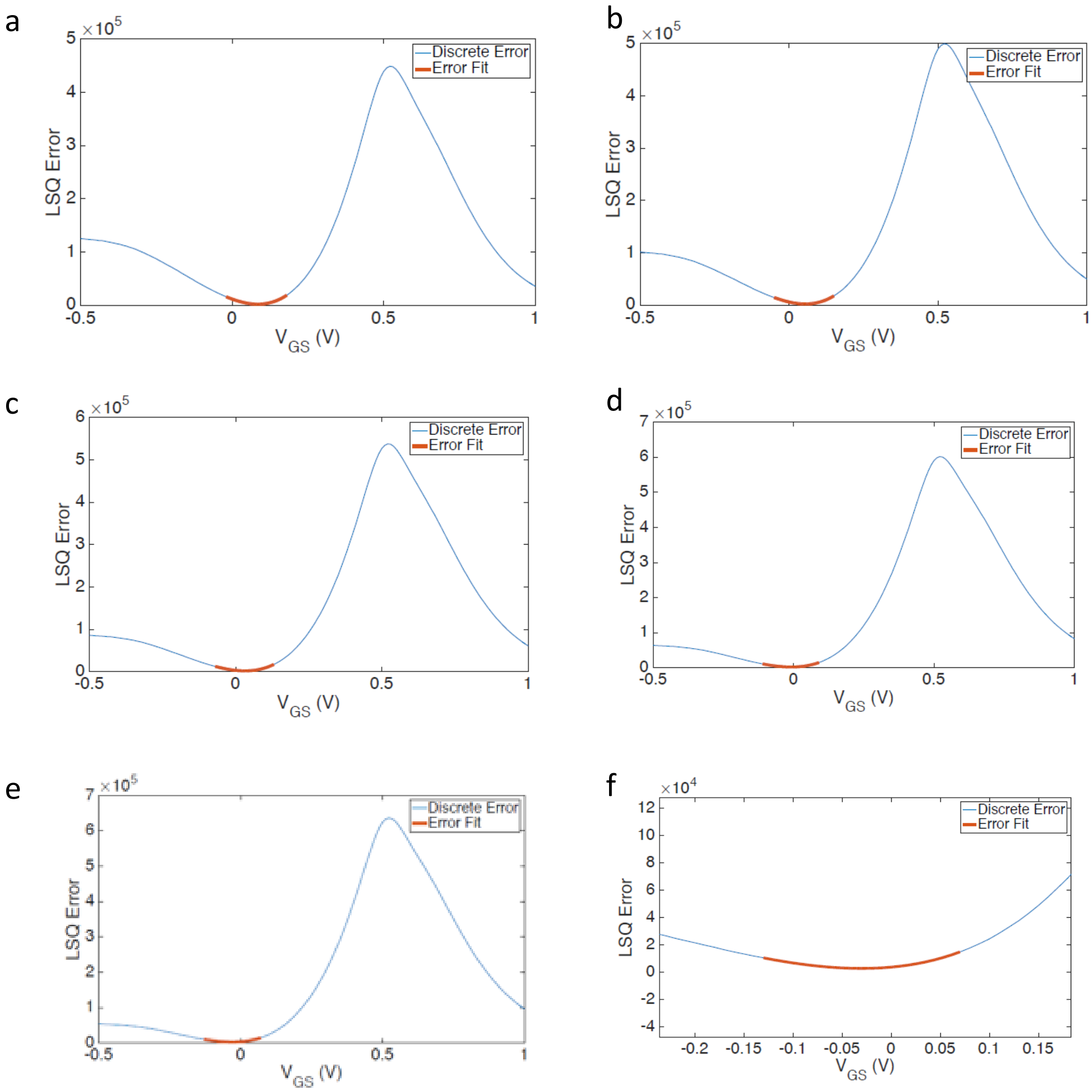

Supplementary Figure 8 | Least Square Error Fit. Least square error for profile matching at calcium ion concentrations **a** 10 µM; **b** 100 µM; **c** 1 mM; **d** 10 mM; **e** 100 mM; and **f** is a zoomed in view of the 10 µM least squares error function showing excellent fit of the 4th-order polynomial.

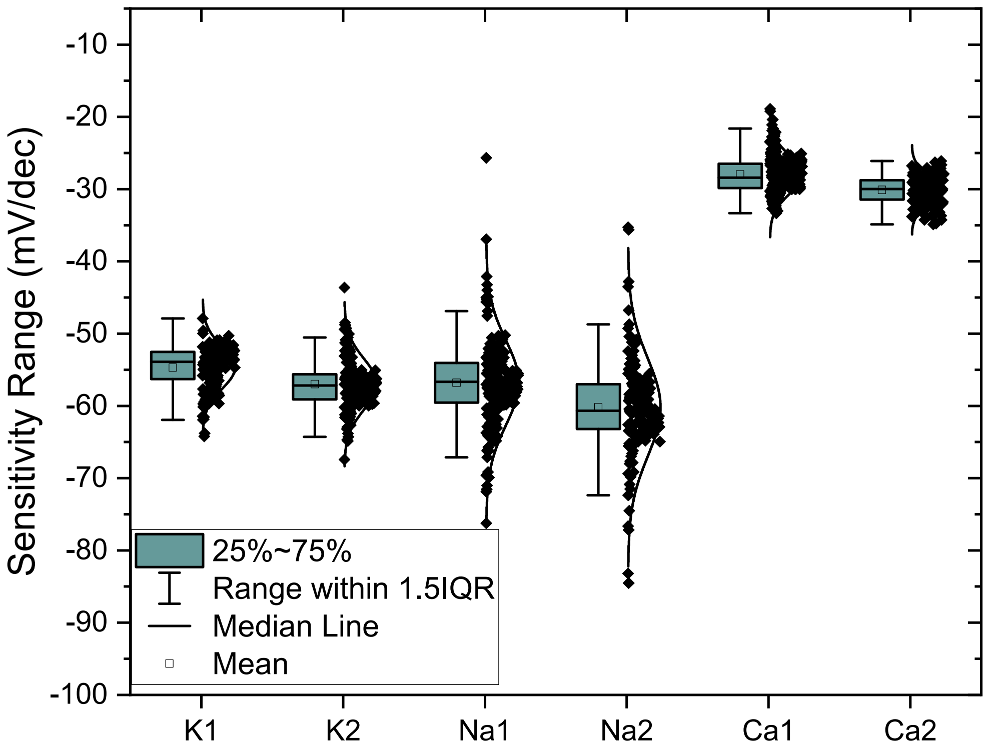

Supplementary Figure 9 | Reproducibility among different chips. Comparison of sensitivity distribution of two batches of sensing chips with different ISMs functionalization. Similar sensor behavior and sensitivity distribution is observed across different sensing chips.

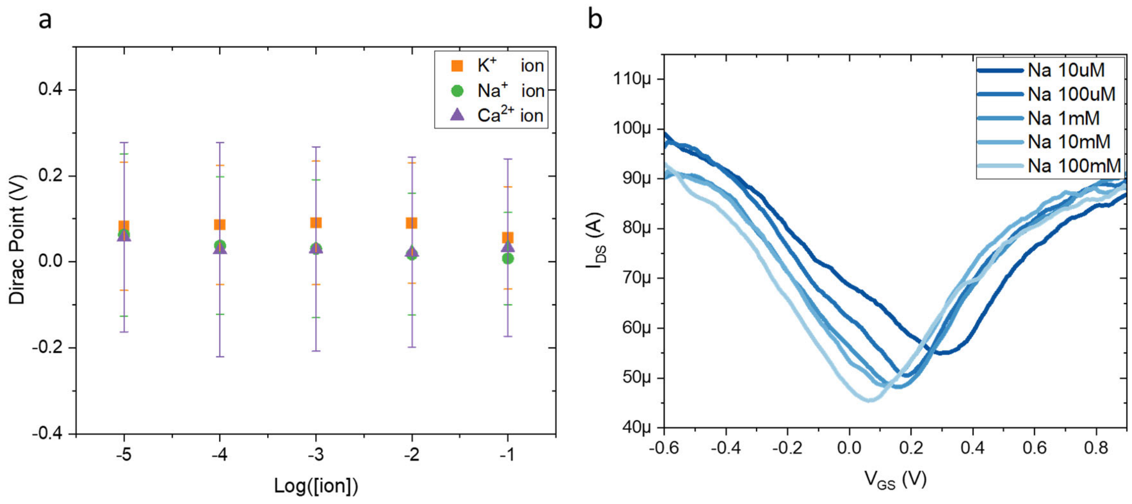

Supplementary Figure 10 | Reponses of as-fabricated graphene sensors. a average Dirac Points shift with respect to changes in K^+, Na^+ and Ca^{2+} ion concentration. Error bar is the standard deviation over multiple devices. **b** shift of *I-V* characteristics of an as-fabricated graphene sensor without functionalization towards changes in Na+ ion concentration.

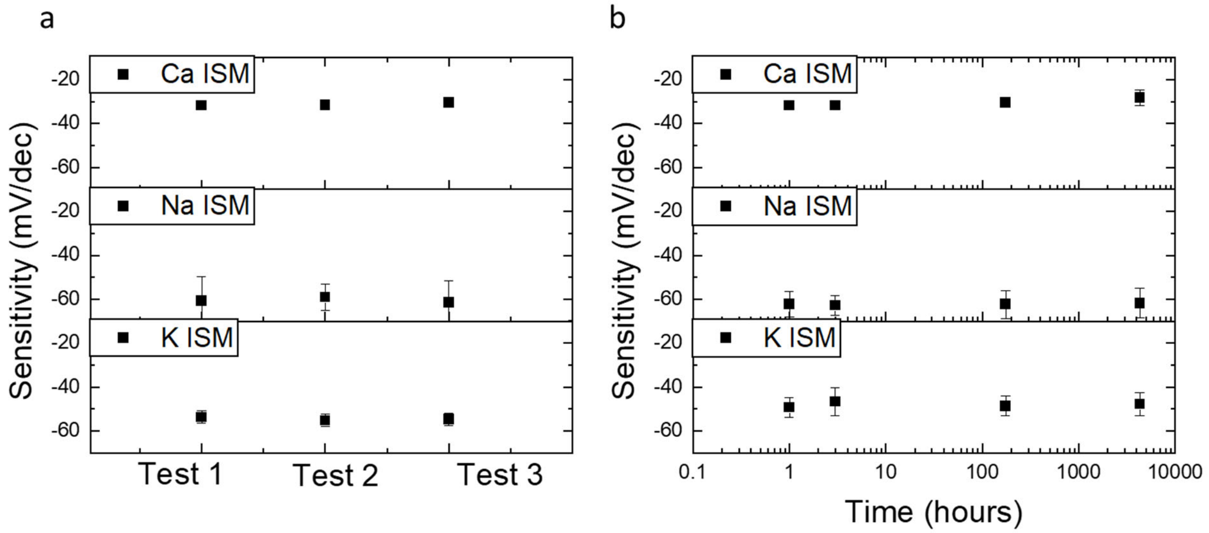

Supplementary Figure 11 | Sensor stability. Sensitivity of K^+, Na^+ and Ca^{2+} ion sensors plotted **a** over multiple measurements and **b** over long period of time (up to 6 months). All

sensors show negligible drift and good stability. Error bars indicate the standard deviation of the sensitivity of all the working devices on the chip.

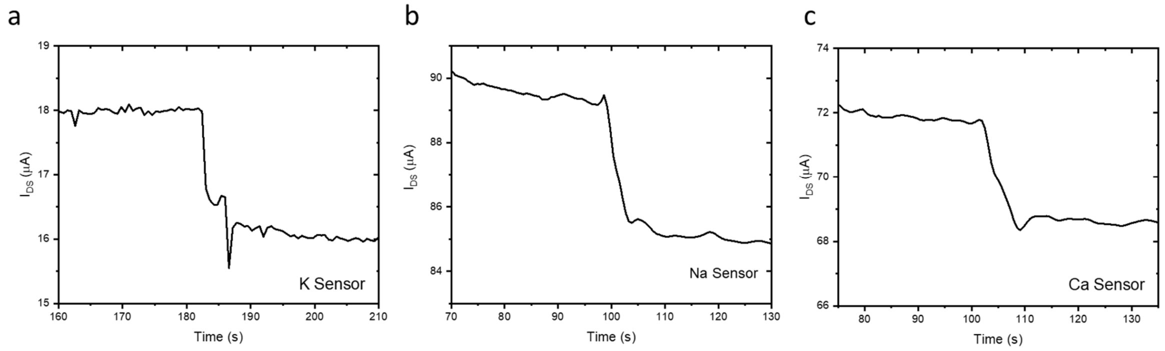

Supplementary Figure 12 | Transient response. Transient response of **a** K^+ ISM, **b** Na^+ ISM, **c** Ca^{2+} ISM functionalized sensor changing to target ion concentration from 10 mM to 100 mM. The response time is calculated by locating the timestamps where current level changes are larger than one standard deviation from its steady-state value. The average response time towards K^+, Na^+, Ca^{2+} ions are 7.4 ± 1.3 s, 5.9 ± 3.3 s, 5.1 ± 1.1 s.

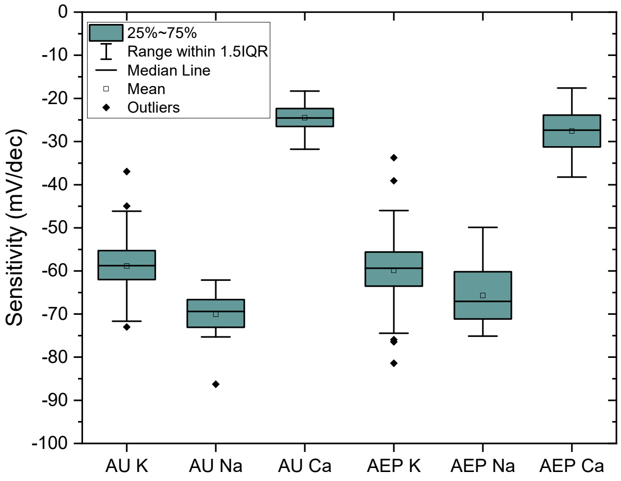

Supplementary Figure 13 | Sensor stability in artificial biofluid. Sensitivity of K^+, Na^+ and Ca^{2+} ion sensors in artificial urine (AU) and artificial eccrine perspiration (AEP).

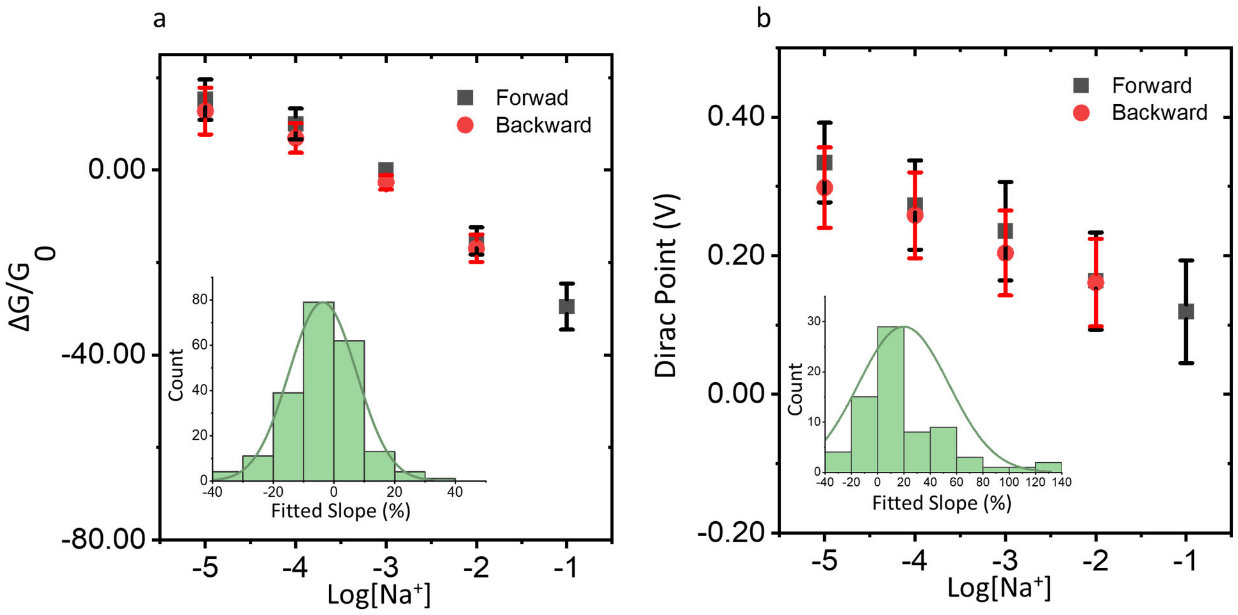

Supplementary Figure 14 | Supplementary reversibility data. The average change in **a** channel conductance and **b** Dirac Point for Na^+ ISM functionalized sensing chip showing excellent reversibility over several magnitude change in Na^+ concentration. Error bars indicate the standard deviation of all the working devices on the chip.

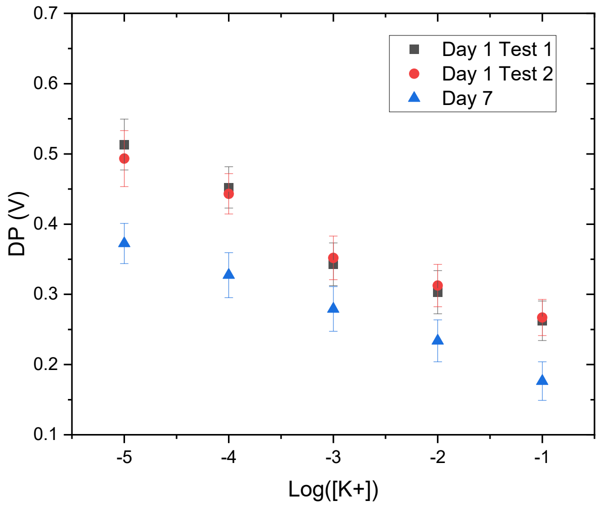

Supplementary Figure 15 | Sensor Calibration: Dirac Point drift of K ISM functionalized sensors over a week showing the necessity of fast and easily calibration schemes. Error bars indicate the standard deviation of the Dirac Points of all the working devices on the chip.

Supplementary Note 4. Least Square Error Fit

Profile matching was used to map the test current slice to a corresponding shift in *I-V* characteristic data. Least squares used to find the optimal shift in *I-V* curve. *I-V* data is discretized in 10 mV increments. To more accurately estimate the optimal shift, the discretized least squares error data was fit near the dirac point (± 100 mV) using a 4th-order polynomial. The minimum of the continuous polynomial function was then used to estimate the optimal shift in *I-V* curve corresponding to the test current slice.

Supplementary Note 5. Synthetic Data Generation

In order to simulate the confidence interval decreasing with increasing sample size, we generated 50,000 synthetic test current responses and *I-V* characteristics based on experimentally observed distributions for this data. Empirical cumulative distribution functions (ECDF) were

computed for the test current data and *I-V* curve data. 50,000 random numbers were then generated from a uniform probability distribution. The uniformly distributed random numbers were then used with inverse transform sampling to randomly generate test current responses according to the ECDF for the transient response data (Fig. 1g). These random test current responses could then be mapped to corresponding *I-V* curve data by performing a weighted of *I-V* curve data according to the location of the test current data on the ECDF. The test current data could then be fit to *I-V* curve data using least squares to determine the necessary shift in *I-V* curve and hence concentration. An outline of the process is depicted in Supplementary Fig. 16a. The distribution for current sensitivity is also provided as a reference in Supplementary Fig. 16b.

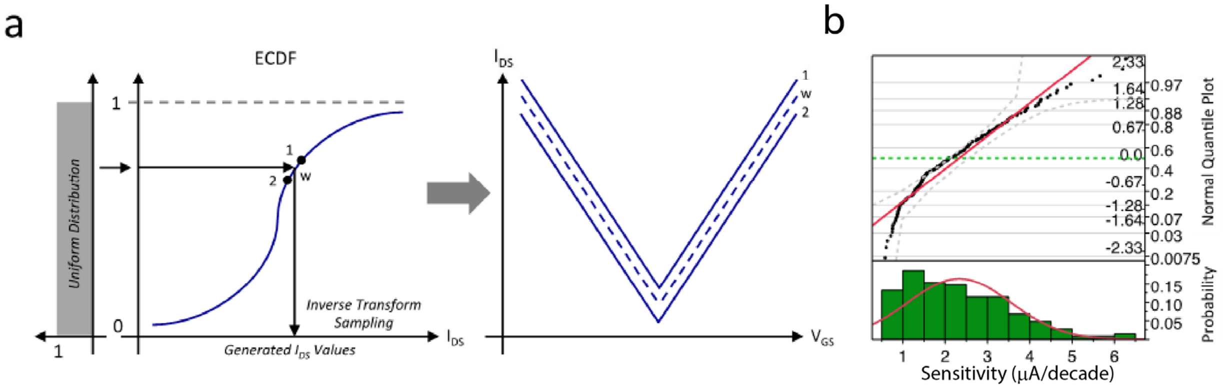

Supplementary Figure 16 | Synthetic Data Generation. a Overview of the process for generating synthetic transient data and *I-V* curve data that is distributed according to experimentally observed data. **b** Probability distribution of sensor sensitivities in μA/decade. Distribution is a skewed Gaussian because sensitivities cannot be negative. Ideal Gaussian (red) with 95% confidence interval (dashed gray) and 50th-percentile (dashed green).

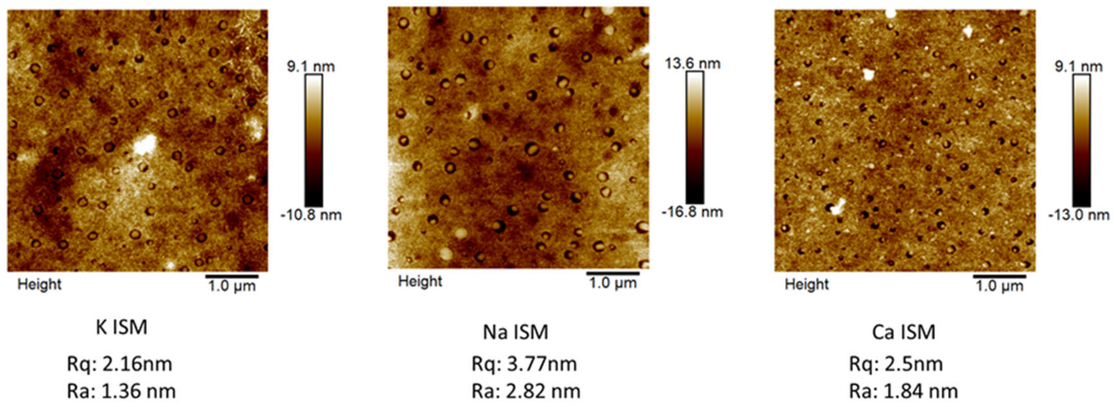

Supplementary Figure 17 | Roughness of printed ISMs. Atomic force microscope of printed ion-selective membranes (ISM) for K^+, Na^+ and Ca^{2+} ions.

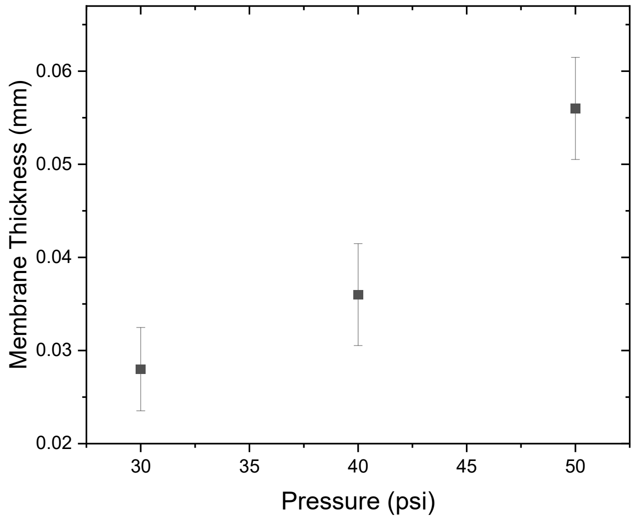

Supplementary Figure 18 | Roughness of printed ISMs: Average membrane thickness over an area of 2.5mm-by 0.5mm printed by the material jetting printer with different pressure. Five measurements were taken with a digital caliper across each membrane strip. Error bars indicate the standard deviation of the five measurements.

Supplementary Notes 6: Ionophore doping effect

We think that one of the reasons that contribute to the more neutral functionalization effect of the Na ISM can attribute to the molecular structure of the Na ISM (Supplementary Fig. 19b). It consists of a calix[4]arene structure that prefers to stay in the "cone" conformation where the ester groups are on the same side[7]. Moreover, calixarenes are believed to interact with graphene surface via hydrophobic interaction and π- π stacking[8]. This would lead to the polar functional group in most of the Na ISM molecules pointing away from the graphene surface, hence resulting in the more neutral functionalization observed. On the other hand, K and Ca ISMs (Supplementary Fig. 19a and Supplementary Fig. 19c) have flatter and more flexible molecular structures that would result in a closer distance between the functional group and the graphene surface, resulting in a more significant doping effect.

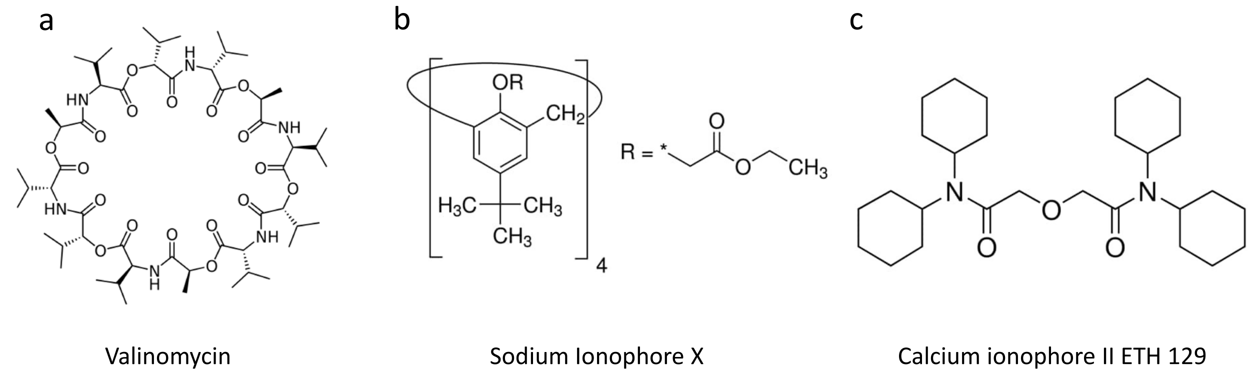

Supplementary Figure 19 | Molecular structure of the ionophore: **a** Valinomycin for K ISM, **b** Sodium ionophore X for Na ISM, **c** ETH129 for Ca ISM.

Supplementary Note 7. Principle Component Analysis (PCA)

PCA is used to reduce the dimensionality of the data collected using the integrated graphene ion sensor array. We first use the Dirac Point as the feature value for the PCA. The PCA matrix e has a dimension of N×M where N is the pure solution set with N = {$K^+_{100\mu M}$, K^+_{1mM}, K^+_{10M}, K^+_{100mM}, $Na^+_{100\mu M}$, Na^+_{1mM}, Na^+_{10M}, Na^+_{100mM}, $Ca^{2+}_{100\mu M}$, Ca^{2+}_{1mM}, Ca^{2+}_{10M}, Ca^{2+}_{100mM}}. The M features are the Dirac Point of individual devices on the sensing chip measured in each pure solution. All three types of the ISMs were integrated onto the sensing chip to provide a multivariate dataset. The labels for the 12 observations are the type of ion that is presents in the pure solution, namely L = {K^+, K^+, K^+, K^+, Na^+, Na^+, Na^+, Na^+, Ca^{2+}, Ca^{2+}, Ca^{2+}, Ca^{2+}}. The matrix was standardized as

$$z(c^l, i) = D(c^l, i) - D(c_0^l, i), l = \{K, Na, Ca\}, i = \{1, 2, \ldots, m\}$$

where $z(c^l, i)$ is standardized value for device i measured in solution c with ion l; D is the raw Dirac Point measurement and c_0^l is a reference solution which in this case is pure K^+, Na^+, and Ca^{2+} solution at 1µM.

The sensitivity towards K^+, Na^+, Ca^{2+} solutions of each individual device can also be used as features for PCA analysis to further separate the classes. The sensitivity is extrapolated as the slope of change in Dirac Point with respect to different concentrations. The PCA matrix has a dimension of 3×M. The PCA is plotted in Supplementary Fig. 20 and the first two principal components cover 100% of the total variance in the dataset. This result demonstrated that the sensitivity profile of the integrated sensing chip is significantly different and can be used to fingerprint ion types. Detailed steps of the PCA process are listed below:

1. Centered the columns of the PCA matrix **X** (with a dimension of N*M, N<<M) by subtracting column mean

2. Performed Singular value decomposition (SVD) on matrix: $\mathbf{X} = \mathbf{USV}^T$

   a. **U**: left singular matrix

   b. **S**: diagonal matrix of singular values corresponding to the estimated principal component

   c. **V**: right singular vector corresponding to the estimated principal directions

3. Calculate the score of the first two principal components by $\mathbf{C} = \mathbf{SV}$

4. Plot the first two principal components

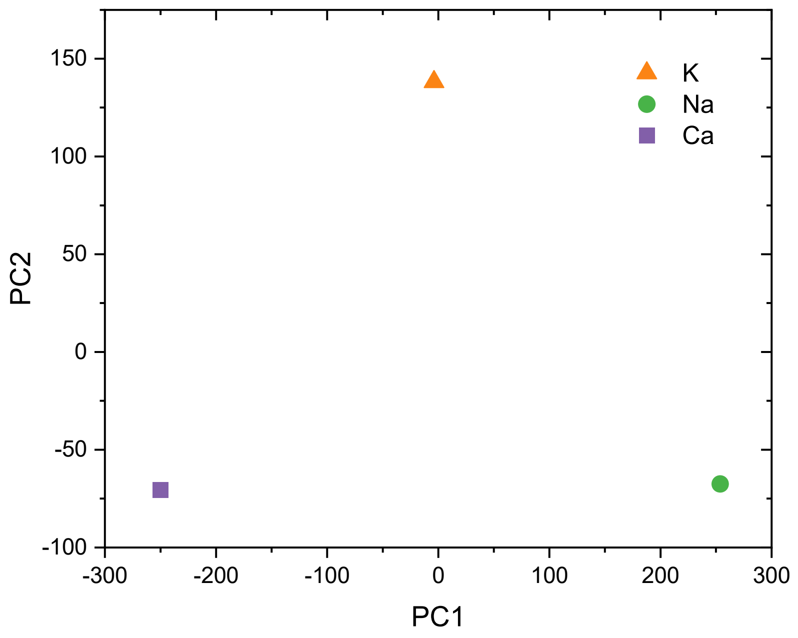

Supplementary Figure 20 | Principal component analysis. First two principal components of chip sensitivity towards electrolytes comprised of different of ions.

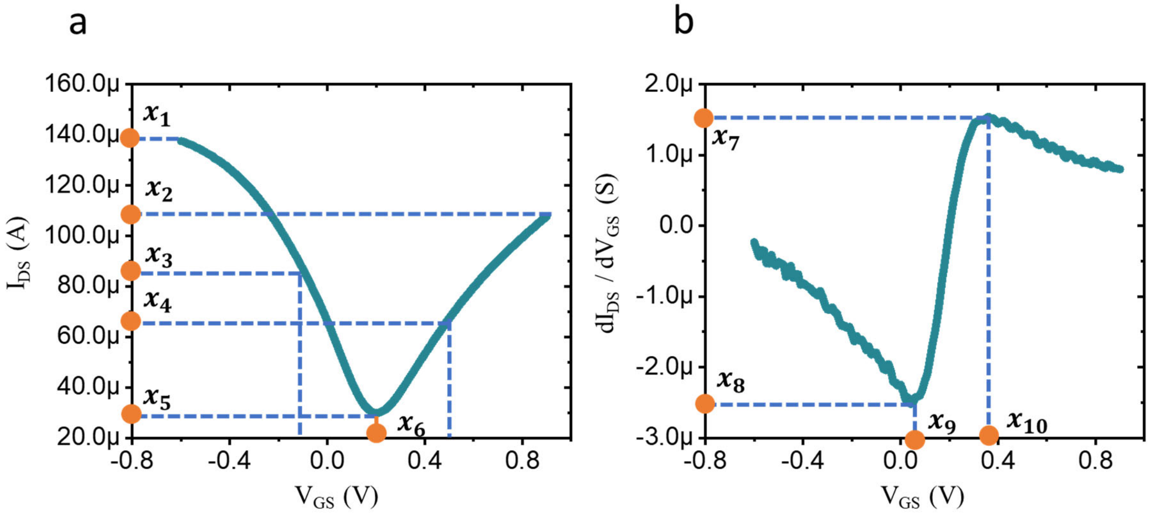

Supplementary Figure 21 | Feature Selection. Measured data showing the selection of feature selection. **a** *I-V* characteristic of GFETs; **b** slope of the *I-V* characteristic. x_1: current at lowest voltage; x_2: current at highest voltage; x_3: current -0.3 V from the lowest point; x_4: current +0.3 V from the lowest point; x_5: current at the Dirac Point; x_6: voltage at the Dirac point; x_7: average of top 6 maximum slope; x_8: average of top 6 minimum slope; x_9: corresponding voltage at x_8; x_{10}: corresponding voltage at x_7.

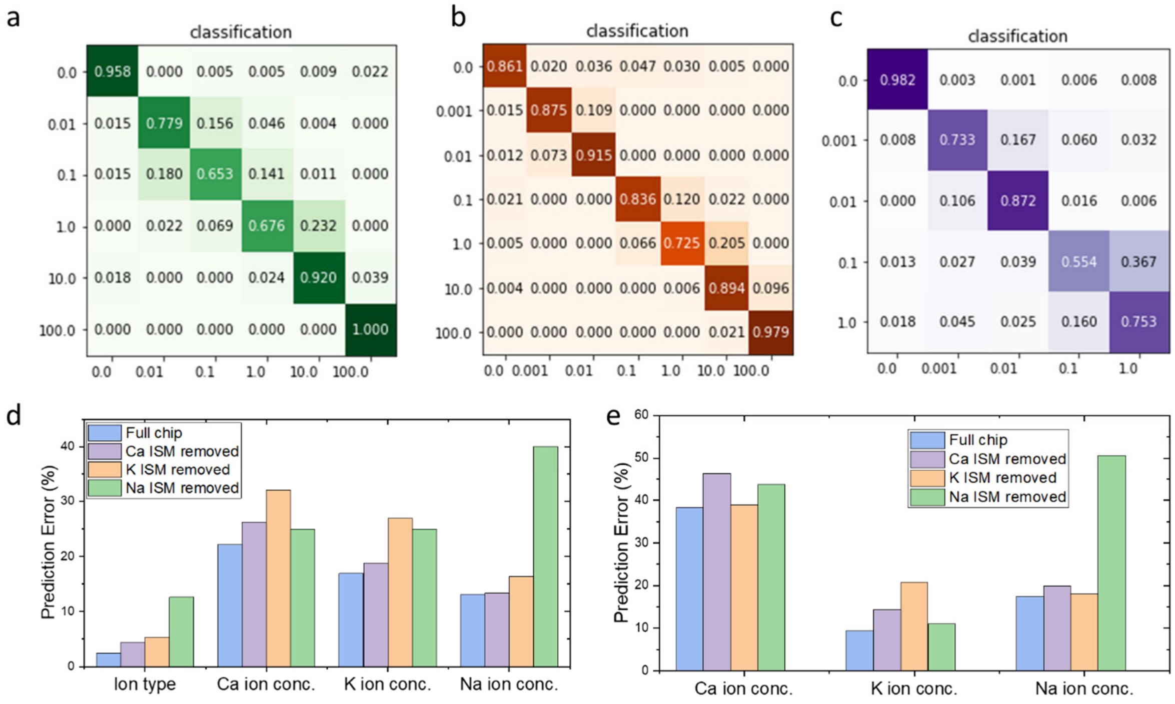

Supplementary Figure 22 | Supplementary Machine Learning Results. Confusion matrix for classification of **a** potassium; **b** sodium, **c** calcium ion in pure solutions using integrated graphene sensing chip. The unit for concentration is mM. Ablation tests show the significance of each functionalization using **d** pure solution and **e** mixture solution.

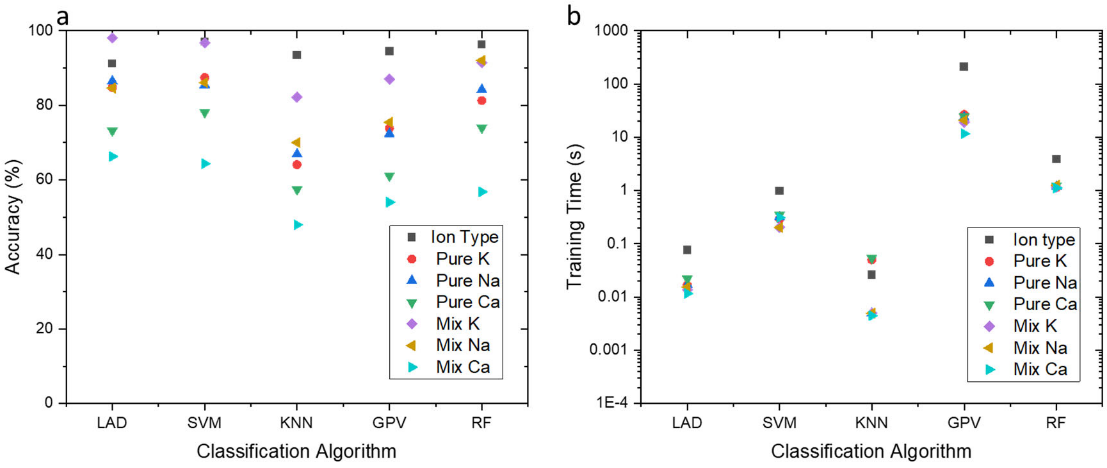

Supplementary Figure 23 | Performance of different algorithm. Comparison of **a** average accuracy and **b** average training time for models trained with Latent Dirichlet Allocation (LAD), Support Vector Machine (SVM), K-Nearest Neighbors (KNN), Gaussian Process (GP) and Random Forest (RF).

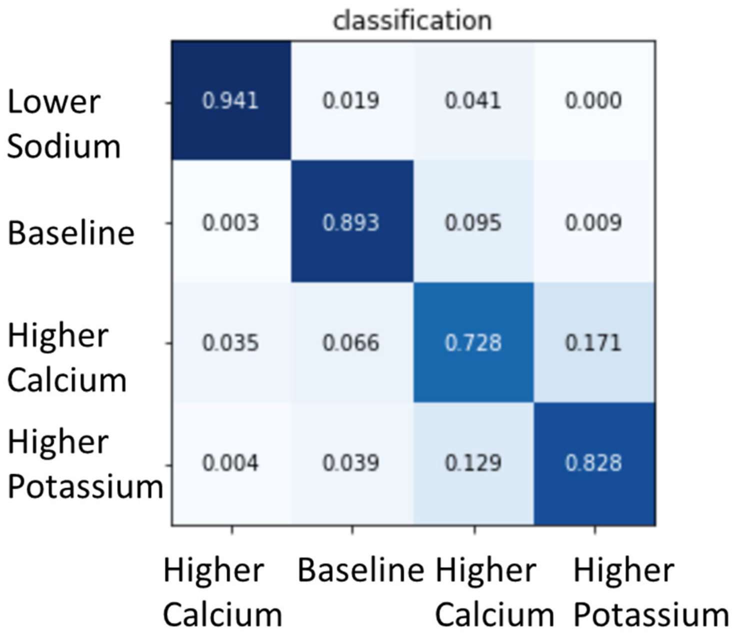

Supplementary Figure 24 | Demonstration in disease diagnosis using the sensor system. Confusion matrix for electrolyte imbalance classification in mixture solutions using integrated graphene sensing chip.

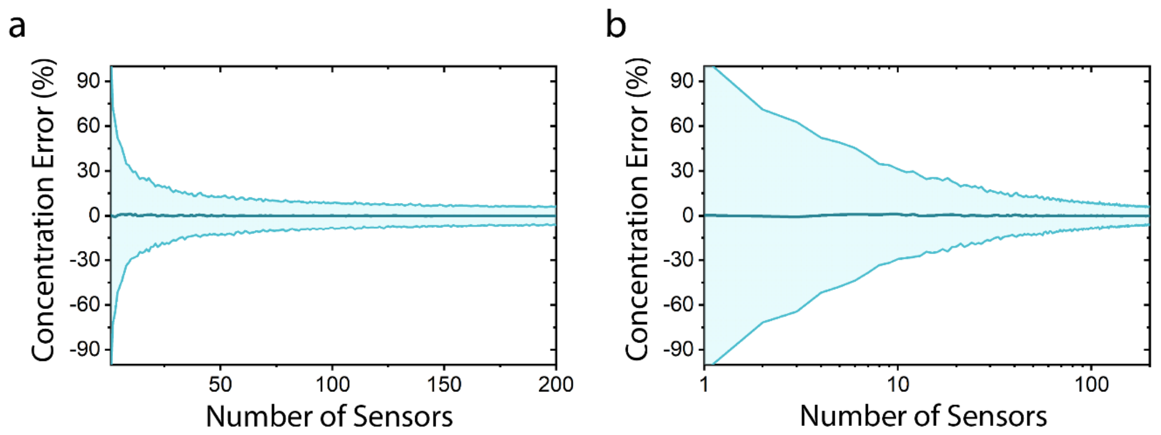

Supplementary Figure 25 | Percent Error of Synthetic Data. Mean percent error and 95% confidence intervals (outer band) as a function of sensor count for ion concentrations in both **a** linear and **b** semi-log scale.

Supplementary Table 1. Performance comparison of ion sensors-based ion-selective membranes

Device type	Materials	Target Ion	Detection Range	Sensitivity [mV/decade]	# of device tested	Stability
ISE	PEDOT:PSS/Au	Ca^{2+}	0.25 mM - 2 mM	32.7±0.981[9]	6	90 mins of continuous measurements
ISE	PEDOT:PSS/Au	Ca^{2+}	1 mM - 10 mM	18.3±1.7[10]	8	2 days of continuous measurements
ISE	Ag/ZnO	Ca^{2+}	100 nM - 10 mM	29.67[11]	-	N/A
ISE	Graphene/ Carbon glass	K^+	30 µM - 100 mM	59.2[12]	-	3 weeks
ISE	Au	Na^+, K^+	10 - 160 mM, 1 - 32 mM	64.2, 61.3 [13]	8	5 weeks
ISFET	Graphene/Au	K^+	10 nM - 1 mM	7.8[14]	1	N/A
Chem-resistor	Self-assembled graphene/Au	Na^+, K^+, Ca^+, H^+	2 - 5 mM	- [15]	1	N/A
ISFET	Graphene/Au	K^+, Na+, Cl^-, etc.	10 µM - 100 mM	49.2, 45.7, −43.0 ± 0.2[16]	4	5 months
Chem-resistor	MoS_2/Au	Na^+,Pb^{2+}, Hg^{2+}, Cd^{2+}	-	- [17]	5	N/A
ISE	LabForce Commercial device	K^+	1nM - 1M	56±3	1	12 months
ISE	LabForce Commercial device	Na^+	4.4nM - 1M	55±3	1	12 months
ISE	LabForce Commercial device	Ca^{2+}	500nM - 1M	26±2	1	12 months
ISFET (this work)	Graphene/Au	K^+, Na^+, Ca^{2+}	10 µM - 100 mM	-54.7 ± 2.90, -56.8 ± 5.87, -30.1 ± 1.90	>200	6 months

Supplementary Table 2. Molecule contents of Biochemazone™ artificial urine (AU) and artificial eccrine perspiration (AEP)

Composition type	Composition name	Artificial Eccrine Perspiration (AEP)	Artificial Urine (AU)
Cation	K^+	Y	Y
	Na^+	Y	Y
	Ca^{2+}	Y	Y
	Mg^{2+}	Y	Y
	NH_4^+	Y	Y
Anion	Cl^-	Y	Y
	PO_4^{3-}		Y
	$C_6H_5O_7^{3-}$ (Citrate ion)		Y
	SO_4^{2-}	Y	Y
	SO_3^{2-}		Y
	HPO_4^{2-}		Y
	$H_2PO_4^-$		Y
Metabolites	Uric Acid	Y	Y
	Urea	Y	Y
	Creatinine		Y
	Lactic Acid	Y	
	Acetic Acid	Y	
	Histidine	Y	

Supplementary Table 3. Pure solution set ion concentration for sensor testing

**Solution name**	**Ca^{2+} ion concentration**	**K^+ ion concentration**	**Na^+ ion concentration**
Pure_Ca_1µM	**1µM**	0	0
Pure_Ca_10µM	**10µM**	0	0
Pure_Ca_100µM	**100µM**	0	0
Pure_Ca_1mM	**1mM**	0	0
Pure_K_10uM	0	**10uM**	0

Pure_K_100uM	0	**100uM**	0
Pure_K_1mM	0	**1mM**	0
Pure_K_10mM	0	**10mM**	0
Pure_K_100mM	0	**100mM**	0
Pure_Na_1µM	0	0	**1µM**
Pure_Na_10µM	0	0	**10µM**
Pure_Na_100uM	0	0	**100µM**
Pure_Na_1mM	0	0	**1mM**
Pure_Na_10mM	0	0	**10mM**
Pure_Na_100mM	0	0	**100mM**

Supplementary Table 4. Mixture solution set ion concentration for sensor testing

**Solution name**	**Ca^{2+} ion concentration**	**K^{+} ion concentration**	**Na^{+} ion concentration**	**Label**
Mix_Ca_100µM	**100µM**	5mM	30mM	-
Mix_Ca_1mM	**1mM**	5mM	30mM	Baseline
Mix_Ca_10mM	**10mM**	5mM	30mM	Higher Calcium
Mix_Ca_100mM	**100mM**	5mM	30mM	Higher Calcium
Mix_K_100µM	1mM	**100µM**	30mM	-
Mix_K_1mM	1Mm	**1mM**	30mM	Baseline
Mix_K_10mM	1mM	**10mM**	30mM	Higher Potassium
Mix_K_100mM	1mM	**100mM**	30mM	Higher Potassium
Mix_Na_100µM	1mM	5mM	**100µM**	Lower Sodium
Mix_Na_1mM	1mM	5mM	**1mM**	Lower Sodium
Mix_Na_10mM	1mM	5mM	**10mM**	Lower Sodium
Mix_Na_100mM	1mM	5mM	**100mM**	Baseline

Supplementary Table 5. Ion sensitive Membrane mixture recipe

	K^+ ISM		Na^+ ISM		Ca^{2+} ISM	
**Chemicals**	weight (g)	wt%	weight (g)	wt%	weight (g)	wt%
**NPOE***	0.661	65.7	0.66	66.1	0.66	62.2
**PVC**	0.33	32.8	0.33	33	0.33	31.1
**KTCB**	0.005	0.99	0.002	0.7	0.06	5.7
**valinomycin**	0.01	0.50	0	0	0	0
**Na X**	0	0	0.007	0.2	0	0
**Ca II**	0	0	0	0	0.01	0.95

*NPOE: 2-Nitrophenyl octyl ether; Na X: Sodium Ionophore X; KTCB: Potassium Tetrakis(4-chlorophenyl) borate; Ca II: Calcium Ionophore II

Supplementary References

1. Schaller, U., Bakker, E. & Pretsch, E. Carrier Mechanism of Acidic Ionophores in Solvent Polymeric Membrane Ion-Selective Electrodes. *Anal. Chem.* **67**, 3123-3132 (1995).

2. Bakker, E., Bühlmann, P. & Pretsch, E. Carrier-based ion-selective electrodes and bulk optodes. 1. General characteristics. *Chem. Rev.* **97**, 3083–3132 (1997).

3. Bakker, E., Bühlmann, P. & Pretsch, E. Carrier-based ion-selective electrodes and bulk optodes. 1. General characteristics. *Chem. Rev.* **97**, 3083–3132 (1997).

4. Jung, S.-H. et al. Super-Nernstian pH Sensor Based on Anomalous Charge Transfer Doping of Defect-Engineered Graphene. *Nano Lett* **21**, 42 (2021).

5. Goodwin, D. G. et al. Faradaic effects in electrochemically gated graphene sensors in the presence of redox active molecules. *Nanotechnology* **31**, 405201 (2020).

6. Zhang, M. et al. High-performance dopamine sensors based on whole-graphene solution-gated transistors. *Adv. Funct. Mater.* **24**, 978–985 (2014).

7. Loon, J. D., Groenen, L. C., Verboom, W., Reinhoudt, D. N. & Wijmenga, S. S. Upper Rim Calixcrowns: Elucidation of the Mechanism of Conformational Interconversion of Calix[4]arenes by Quantitative 2-D EXSY NMR Spectroscopy. *J. Am. Chem. Soc.* **113**, 2378–2384 (1991).

8. Eroglu, E. et al. Nitrate uptake by p-phosphonic acid calix[8]arene stabilized graphene. *Chem. Commun.* **49**, 8172–8174 (2013).

9. Nyein, H. Y. Y. et al. A Wearable Electrochemical Platform for Noninvasive Simultaneous Monitoring of $Ca^{2+}$and pH. *ACS Nano* **10**, 7216–7224 (2016).

10. Lee, Y. K. et al. Chemical Sensing Systems that Utilize Soft Electronics on Thin Elastomeric Substrates with Open Cellular Designs. *Adv. Funct. Mater.* **27**, 1605476 (2017).

11. Asif, M. H. et al. Functionalized zinc oxide nanorod with ionophore-membrane coating as an intracellular Ca^{2+} selective sensor. *Appl. Phys. Lett.* (2009).

12. Li, F. et al. All-solid-state potassium-selective electrode using graphene as the solid contact. *Analyst* **137**, 618–623 (2012).

13. Gao, W. et al. Fully integrated wearable sensor arrays for multiplexed in situ perspiration analysis. *Nature* **529**, 509–514 (2016).

14. Maehashi, K. et al. Selective ion sensors based on ionophore-modified graphene field-effect transistors. *Sensors and Actuators B Chem.*, **187**, 305–308 (2020).

15. Zhang, B. & Cui, T. High-perfermance and low-cost ion sensitive sensor array based on self-assembled graphene. *Sensors Actuators A Phys.* **177**, 110–114 (2012).

16. Fakih, I. et al. Selective ion sensing with high resolution large area graphene field effect transistor arrays. *Nat. Commun.* **11**, 1–12 (2020).

17. Li, P., Zhang, D. & Wu, Z. Flexible MoS2 sensor arrays for high performance label-free ion sensing. *Sensors Actuators A Phys.* **286**, 51–58 (2019).
